# Associations between lifestyle, physical and social environments and frailty among Chinese older people: a multilevel analysis

**DOI:** 10.1186/s12877-018-0982-1

**Published:** 2018-12-14

**Authors:** Bo Ye, Junling Gao, Hua Fu

**Affiliations:** 1 School of Public Health, Fudan University, Shanghai, China; 2 Fudan Health Communication Institute, School of Public Health, Fudan University, PO Box 248, 138 Yixueyuan Road, Shanghai, 200032 China

**Keywords:** Frailty, Lifestyle, Physical environment, Social environment, Multilevel modelling

## Abstract

**Background:**

Frailty represents a public health priority and an increasingly prevalent condition in the ageing population. It is seen as reflecting an interaction among individual factors and a range of environmental elements. This study aims to examine the association between frailty and individual factors, physical and social environments among Chinese older people.

**Methods:**

The data were from the Shanghai Healthy City Survey in 2017, which sampled 2559 older people aged ≥60 years from 67 neighbourhoods. The FRAIL scale was used to assess frailty, and social and physical environments were assessed using validated and psychometrically tested instruments. Individual factors included age, gender, education, employment, marital status, smoking, drinking, physical exercise, organization participation, self-rated health and psychological well-being. A multilevel analysis was conducted to examine whether physical and social environments were associated with frailty.

**Results:**

The prevalence of pre-frailty and frailty were 39.5 and 16.9%, respectively. The prevalence of frailty increased with age from 14.6% (60–64 years) to 26.5% (≥75 years). After adjusting for age and/or gender, older age, women, and those with low education, alcohol dependence, physical inactivity, poor self-rated health, or psychological disorders had a higher prevalence of frailty. The multilevel analysis indicated that after controlling for individual covariates, compared to the 1st quartile of aesthetic quality, the odds ratio (OR) of frailty for the 4th quartile was 0.65 (0.47–0.89); compared to the 1st quartile of walking environment, the OR of frailty for the 4th quartile was 0.43 (0.19–0.95); compared to the 1st quartile of social cohesion, the OR of frailty for the 4th quartile was 0.73 (0.54–0.99); compared to the 1st quartile of social participation, the ORs of frailty for the 2nd, 3rd and 4th quartiles were 0.76 (0.59–0.97), 0.59 (0.45–0.77) and 0.59 (0.45–0.77), respectively.

**Conclusions:**

Frailty is a highly prevalent health condition among the aged population in China. Healthcare should focus on frail elderly who are older age, women, those with low education, and those with mental health problems. It may decrease frailty among Chinese older people to encourage social participation and healthy behaviours and to build aesthetic, walkable and cohesive neighbourhoods.

**Electronic supplementary material:**

The online version of this article (10.1186/s12877-018-0982-1) contains supplementary material, which is available to authorized users.

## Background

Frailty increases an individual’s vulnerability to increased dependency and death [1]. Numerous studies have shown that frailty often leads to a higher risk of worsening disability, falls, hospital admissions, and mortality [2]. The prevalence of frailty among community-dwelling older people in the Asia-Pacific region is approximately 3.5–27%, which is comparable to the prevalence across Europe and the Americas [3, 4]. Frailty increases with age [5], it represents a public health priority for multiple reasons, and it is a highly and increasingly prevalent condition in the ageing population [4]. Importantly, frailty may be even more prevalent in low- and middle-income countries [6]. Thus, the frailty among older people in China, which is a developing country with the largest and most rapidly growing ageing population, would be a more serious and urgent public health issue.

A large amount of evidence shows that multiple aetiologic factors, including cumulative cellular damage, malnutrition, sarcopenia, and inflammation, contribute to frailty [7]. Exercise and appropriate nutrition are first-line treatments for frailty [2]. From a public health perspective, lifestyle and healthy behaviours such as physical activity and a healthy diet seem to play important roles in preventing frailty. Previous studies also indicated that physical inactivity and alcohol use were positively related to frailty [8, 9]. Other factors linked with frailty development include socio-demographic characteristics, such as poverty, educational level and marital status [8–10]. One previous study indicated older people who participated in organizations had a lower risk of functional disability [11]. Moreover, the lifestyle of older people are probably affected by neighbourhood environments where they live. For example, transportation, social activities and healthy diet behaviours were proved to be related to neighbourhood walkability and aesthetics [12, 13]. It is important to understand the associations between lifestyle and frailty in the local context.

According to theories of environmental gerontology, over their life span, people are influenced by an ongoing interchange between the individual and their social and physical environment [14]. Frailty is seen as reflecting an interaction among individual factors and a range of environmental elements [15]. Recently, the WHO proposed a new goal about healthy ageing, which redefines the process of developing and maintaining the functional ability that enables well-being in older age [6]. It highlights the influence of intrinsic capacity and the relevant environment. Frailty is a complex health issue and commonly known as a geriatric syndrome [16]. It is not just the result of a decline in intrinsic capacity but is more affected by the living environment. For example, those who have declined mobility would be most limited by not having walkable roads or accessible facilities or emotional and practical support, then would become frail, in a vicious circle. Increasing physical activity has been showed to be an effective intervention for frail elderly people [17]; meanwhile, physical activity is most influenced by the neighbourhood environment. It is reasonable to believe that the living environment plays an important role in the transformation of frailty status. Especially for many older people, the neighbourhood of residence represents their predominant environmental context.

In recent years, China has been undergoing a rapid transition from a rural to an urban society, meanwhile facing the largest and the most rapidly growing ageing population in the world [18]. Shanghai is one of the most rapidly ageing cities in China, with 14.3% of the population being older adults aged 65 and above, and the proportion is expected to increase to nearly 20% by 2030 [19]. Great changes have taken place in Shanghai’s construction, such as rural relocation and community reconstruction. The physical environment of community has changed, and some people from different areas have moved into new communities. The environmental characteristics of neighbourhood are more important to older people who are likely to be spending more time with neighbours in their immediate neighbourhood [20]. However, there are few studies on physical environment and frailty. Most studies focus on the relationships between physical environment and behaviours and/or well-being among older people [21–23]. The social environment is also important for preventing or reducing frailty, and different dimensions of the social environment have different effects [24]. However, the correlation between frailty and the dimensions of the social environment, such as social support and social network are discovered inconsistent results [24]. Another study indicated that social cohesion, neighbourhood belonging and feeling secure were protective factors against frailty [25]. We believe it is necessary to examine the relationships between physical and social neighbourhood environments and frailty based on the rare evidence that has been presented.

The aims of the present study were: 1) to examine the individual-level socio-demographic, lifestyle, and health condition correlates of frailty; and 2) to examine the relationships between individual- and neighbourhood-level physical and social environments with frailty among Chinese older people.

## Methods

### Participants and study design

In shanghai, 8089 community-living residents were recruited in the Shanghai Healthy City Survey (Round 5) from June to September 2017 using a multistage stratified random sampling method. First, we used stratified random sampling to select neighbourhoods/villages in each district. Second, we used simple random sampling to select residents’ addresses in each selected neighbourhood/village. Lastly, the trained health-related workers from each selected neighbourhood/village committee visited the addresses and interviewed residents using uniform questionnaires after obtaining the informed consent in person. In addition, if there was no respondent in the house after two visits or the worker was rejected, we could investigate their neighbours. Inclusion criteria were: 1) live at the address for more than half a year; 2) age 15 years and older; and 3) birthday is closer to the survey date. Exclusion criteria were: 1) severe psychological disorders; and 2) inability to answer questions. The Ethics Committee for Medical Research at the School of Public Health, Fudan University approved the study.

We used a sub-sample (2846/8089, 35.2%) of subjects who were 60 years of age or older. In Shanghai, the neighbourhoods were clustered administratively. Specifically, every sub-district of a city’s district administers many neighbourhoods. Each neighbourhood has a committee to administer the residents living in that neighbourhood [26]. In total, 2559 (89.9%) older people were sampled from 67 neighbourhoods (mean = 38 elders; range: 2–104 elders) after excluding the incomplete data (Additional file 1: Sheet 1).

### Measurements

#### Frailty

The FRAIL scale was used to measure frailty. It consists of 5 items (Fatigue, Resistance, Ambulation, Illness, and Loss of weight) from both the Frailty Index (FI) and Freid’s Frailty Phenotype (FP) [27]. The FRAIL scale was a brief and valid tool that could effectively identify frailty/pre-frailty status, as well as had a strong ability to predict mortality risk [28]. Meanwhile, a previous study showed that the FRAIL scale could be used by non-healthcare professionals as a community screening tool for frailty among Chinese older people [29]. Frailty scores range from 0 to 5 (i.e., 1 point for each component; 0 = best to 5 = worst) and represent frail (3–5), pre-frail (1–2), and robust (0) health status.

### Environmental characteristics

#### Physical environment

Two physical characteristics of neighbourhood were included in our study. One of the physical characteristics of neighbourhood is aesthetic quality (AQ), which consists of 5 items with item 1 and item 2 being reverse coded. The other physical characteristics of neighbourhood is walking environment (WE), which consists of 7 items. All response categories were ‘strongly disagree’, ‘disagree’, ‘neutral’, ‘agree’, and ‘strongly agree’ on a scale of 1 to 5. Mujahid et al. [30] developed the original scale, and the Chinese version was revised and validated [23]. Cronbach’s alpha of the two scales were 0.75 and 0.91 for the current sample, respectively.

#### Social environment

Two social characteristics of neighbourhood were also included. One of social characteristics of neighbourhood is social cohesion (SC), which comes from Mujahid et al. [30], and consists of 4 items. Each item also was assessed using a five-point Likert scale, ranging from ‘strongly disagree’ (1) to ‘strongly agree’ (5). The other social characteristics of neighbourhood is social participation (SP), which was developed by Gao et al. and was validated in Chinese populations [22, 23]. The scale is assessed by asking respondents how often in the past 12 months did you participate in the eight types of social activities (see Table 1). The answer for each social activity were ‘never’, ‘several times per year’, ‘several times per month’, ‘once per week’, and ‘two or more times per week’ on a scale of 1 to 5. Cronbach’s alpha of the two scales were 0.91 and 0.84 for the current sample, respectively.

**Table 1:** The eight types of social activities for measuring social participation (SP)

1) visiting family or friends	
2) recreational activities involving other people	
3) physical and cultural activities in the neighbourhood	
4) attending a series of lectures in the neighbourhood	
5) a self-management group or mutual-help group	
6) volunteer or charity work	
7) activities of political organizations or associations	
8) dining out or shopping with other people	

The mean score of individual’s assessment on each scale’s items represented individual-level environmental characteristic. Due to different individuals having varying perceptions of the same reality, using the average of the responses across multiple persons within a neighbourhood reduces measurement error [23, 30]. Therefore, the mean score of all respondents in the same neighbourhood for each scale was used to estimate the neighbourhood-level environmental characteristics. However, there was a limitation that some neighbourhoods only included very small samples [30]. For that reason, we excluded the neighbourhoods with fewer than 30 samples in the multilevel model. Finally, a sample of 2154 (84.2%) elders from 42 neighbourhoods (mean = 51; range: 30–104) was included in multilevel model (Additional file 1: Sheet 2). For analyses, we transformed the mean scores of environmental characteristics into quartiles, with the first quartile indicating the lowest level of environmental characteristics.

#### Individual characteristics

##### Demographic

Demographics included age (age groups were divided into 60–64 years, 65–69 years, 70–74 years, and ≥ 75 years), gender (men and women), educational level (illiteracy, primary school, junior high school, senior high school, and university), marital status (married and other, which included divorced, widowed and unmarried), work status (employed and other, which included retired and unemployed).

##### Lifestyle

Individual lifestyles included smoking, alcohol consumption, moderate intensity physical activity (MIPA), and organization participation. Smoking status was divided into nonsmoker, ex-smoker, and current smoker. Alcohol consumption was divided into 3 categories: nondrinker, drinker, and alcohol dependence. Alcohol dependence was assessed using the CAGE (Cut down, Annoyed, Guilty, Eye opener) questionnaire [31]. The CAGE questionnaire contains 4 items, and a two-item positive response was considered as alcohol dependence in our study. MIPA was assessed using two questions: “Q1. How many times did you participate in moderate intensity physical activity (heart rate and breathing rate increase and slight perspiration) per week? (none, 1-2 times, 3-4 times, 5-6 times, 7 times or more); Q2. How long did you participate every time? (less than 20 min, 20-30 min, 30-40 min, 40-50 min, or more than 50 min).” MIPA was also divided into 3 categories: none, low, and high. Those who answered “none” for Q1 were classified as none; those who answered “3–4 times” or more for Q1 and “30–40 min” or more for Q2 were classified as high; those who answered less than “3–4 times” for Q1 or less than “30–40 min” for Q2 were classified as low. Organization participation was assessed by asking respondents if they participated in six types of organizations: 1) interest activity group (e.g., senior university, senior centre), 2) volunteer organization, 3) government departments (e.g., neighbourhood committees, street offices), 4) party grouping (e.g., party branch, other democratic parties), 5) work-related organization (e.g., worker union, commission of retirees, trade association), or 6) religious organization. Those who participated in any one of these organizations were classified as reporting organization participation (yes), and those who did not participate in any organizations were classified as not reporting organization participation (no).

#### Health condition

Health conditions included self-rated health (SRH) and psychological well-being (PWB). SRH was assessed by a single question: “Would you say that in general your health is excellent, very good, good, fair, or poor?” From this question, we classified the answer as a binary variable (0 = poor, included fair or poor; 1 = good, included excellent, very good, and good). PWB was assessed using a Chinese version of the World Health Organization Well-Being Index (WHO-5), the total score ranges from 0 to 25, and the higher score means a better quality of life. Those who scored lower than 13 or had a score 0 or 1 on any one of 5 items were considered as having a psychological disorder [32].

### Statistical analysis

First, we used descriptive analysis and a χ^2^ trend test to show the prevalence of frailty by gender and age groups.

Second, we used analysis of variance (ANOVA) for continuous variables and χ^2^ test for categorical variables to compare the difference in the prevalence of frailty between groups (robust, pre-frail, frail). Then, an ordinal logistic regression analysis was performed to examine the associations between individual demographics, lifestyle, health conditions (independent variables) and frailty (dependent variable) adjusted for gender and/or age (control variable).

Third, we used a multilevel analysis to explain the relationship between environmental characteristics and frailty. Our final data included 42 neighbourhoods and 2154 elders living in these neighbourhoods. As the individuals (level 1) are nested in neighbourhoods (level 2), a multilevel analytic approach was adopted. We fitted the data using a multilevel mixed-effects ordered logistic regression model, controlling for both individual-level and neighbourhood-level variables as fixed effects and allowing for a random intercept for frailty. After examining the neighbourhood-level variance in frailty without adding any explanatory variables (null model), we successively examined the association between individual-level and neighbourhood-level environmental characteristics and frailty. Then, with controlling for individual covariates, both individual- and neighbourhood-level environmental variables were added to the final model. We estimated the adjusted ORs and their 95% confidence intervals (CIs) of environmental variables for frailty and used − 2 log likelihood (−2LL) and Akaike information criterion (AIC) to compare the goodness-of-fit of each model. The windows-based SPSS version 22.0 (SPSS Inc., Chicago, IL, USA) was used for the descriptive analysis, univariate analysis and logistic regression analysis, and Stata version 14.1 (StataCorp, Texas, USA) was used for the multilevel analysis. A *P* value less than 0.05 was considered statistically significant for all statistical analyses.

## Results

### Prevalence of frailty by age and gender

Two thousand five hundred and fifty-nine community residents aged 60 years and older were incorporated into the final sample. The mean age of total elders was (66.12 ± 4.85) years; 42.6% (*n* = 1089) were men and 57.4% (*n* = 1470) were women. The prevalence of frailty by age and gender is shown in Table 2. The overall prevalence of pre-frailty and frailty was 39.5 and 16.9%, respectively. The prevalence of frailty increased with age, being 14.6% for respondents aged 60–64 years and 26.5% for those aged 75 years and older (*P* trend of < 0.001).

**Table 2:** The Prevalence of Robust, Pre-frail and Frail among Older People by Gender and Age Groups (*N* = 2559)

Frailty status	Gender	Age Group, n (%)					*P trend*
		60-64y	65–69y	70-74y	≥75y	All Ages	
	Both Gender						
Robust (0)		545 (49.2)	369 (41.1)	153 (34.9)	47 (40.2)	1114 (43.5)	
Pre-frail (1–2)		400 (36.1)	382 (42.6)	191 (43.6)	39 (33.3)	1012 (39.5)	
Frail (≥3)		162 (14.6)	146 (16.3)	94 (21.5)	31 (26.5)	433 (16.9)	< 0.001
	Man						
Robust (0)		225 (52.2)	178 (52.2)	73 (36.0)	25 (39.7)	501 (46.0)	
Pre-frail (1–2)		144 (33.4)	157 (40.1)	86 (42.4)	17 (27.0)	404 (37.1)	
Frail (≥3)		62 (14.4)	57 (14.5)	44 (21.7)	21 (33.3)	184 (16.9)	< 0.001
	Woman						
Robust (0)		320 (47.3)	191 (37.8)	80 (34.0)	22 (40.7)	613 (41.7)	
Pre-frail (1–2)		256 (37.9)	225 (44.6)	105 (44.7)	22 (40.7)	608 (41.4)	
Frail (≥3)		100 (14.8)	89 (17.6)	50 (21.3)	10 (18.5)	249 (16.9)	< 0.001

### Individual correlates of frailty

The individual characteristics of older people and factors associated with the presence of frailty are shown in Table 3. Overall, the univariate analysis showed that the majority of individual factors were associated with frailty except for gender, smoking and organization participation.

**Table 3:** Individual Factors associated with Frailty among Older People (N = 2559)

Variable	Mean ± SD/ n (%)					Age- and Gender-adjusted		
	Overall	Robust	Pre-frail	Frail	*P*	OR	95%CI	*P*
Age, year	66.12 ± 4.85	65.53 ± 4.70	66.34 ± 4.64	67.16 ± 5.49	< 0.001	1.05	1.04–1.07	< 0.001^e^
Gender					0.063			
Man	1089 (42.6)	501 (46.0)	404 (37.1)	184 (16.9)		1.00		
Woman	1470 (57.4)	613 (41.7)	608 (41.4)	249 (16.9)		1.17	1.01–1.36	0.039^f^
Education					< 0.001			
Illiteracy	98 (3.8)	25 (25.5)	43 (43.9)	30 (30.6)		1.00		
Primary school	397 (15.5)	177 (44.6)	144 (36.3)	76 (19.1)		0.51	0.34–0.78	0.002
Junior high school	1006 (39.3)	445 (44.2)	383 (38.1)	178 (17.7)		0.54	0.36–0.79	0.002
Senior high school	709 (27.7)	325 (45.8)	286 (40.3)	98 (13.8)		0.47	0.31–0.69	< 0.001
University	349 (13.6)	142 (40.7)	156 (44.7)	51 (14.6)		0.52	0.34–0.79	0.002
Work status					0.001			
Employed	331 (12.9)	169 (51.1)	99 (29.9)	63 (19.0)		0.85	0.69–1.06	0.146
Other^a^	2228 (87.1)	945 (42.4)	913 (41.0)	370 (16.6)		1.00		
Marital status					0.001			
Married	2269 (88.7)	1001 (44.1)	906 (39.9)	362 (16.0)		0.80	0.63–1.00	0.051
Other^b^	290 (11.3)	113 (39.0)	106 (36.6)	71 (24.5)		1.00		
Smoking					0.410			
Nonsmoker	2070 (80.9)	894 (43.2)	815 (39.4)	361 (17.4)		1.00		
Ex-smoker	81 (3.2)	33 (40.7)	32 (39.5)	16 (19.8)		1.20	0.78–1.83	0.407
Current Smoker	408 (15.9)	187 (45.8)	165 (40.4)	56 (13.7)		0.99	0.79–1.24	0.925
Drinking					0.001			
Nondrinker	2057 (80.4)	902 (43.9)	822 (40.0)	333 (16.2)		1.00		
Drinker	359 (14.0)	157 (43.7)	145 (40.4)	57 (15.9)		1.13	0.90–1.41	0.289
Alcohol Dependence	143 (5.6)	55 (38.5)	45 (31.5)	43 (30.1)		1.84	1.33–2.55	< 0.001
MIPA level					< 0.001			
None	753 (29.4)	279 (37.1)	306 (40.6)	168 (22.3)		1.00		
Low	1199 (46.9)	520 (43.4)	480 (40.0)	199 (16.6)		0.74	0.63–0.88	0.001
High	607 (23.7)	315 (51.9)	226 (37.2)	66 (10.9)		0.52	0.43–0.64	< 0.001
Organization Participation					0.241			
Yes	1689 (66.0)	739 (43.8)	679 (40.2)	271 (16.0)		0.89	0.76–1.04	0.136
No	870 (34.0)	375 (43.1)	333 (38.3)	162 (18.6)		1.00		
Self-Rated Health					< 0.001			
Good	1452 (56.7)	803 (55.3)	512 (35.3)	137 (9.4)		1.00		
Poor	1107 (43.3)	311 (28.1)	500 (45.2)	296 (26.7)		3.16	2.70–3.68	< 0.001
WHO-5^c^, score	18.82 ± 4.35	20.14 ± 3.56	18.37 ± 4.41	16.47 ± 4.82	< 0.001	0.87	0.86–0.89	< 0.001
Psychological Disorder^c, d^					< 0.001			
yes	258 (10.1)	38 (14.7)	127 (49.2)	93 (36.0)		3.69	2.88–4.72	< 0.001
no	2295 (89.7)	1072 (46.7)	885 (38.6)	338 (14.7)		1.00		

*SD* standard deviation, *MIPA* moderate intensity physical activity

^a^Including unemployed and retired; ^b^Including divorced, widowed and unmarried; ^c^There were six missing data; ^d^Whichever one item score < =1 or total score < 13 considered psychological disorder; ^e^Only gender was adjusted for age; ^f^Only age was adjusted for gender

Further multivariate analysis indicated that after adjusting for both age and/or gender, the trend of frailty increasing with age remained (adjusted *P* < 0.001). The prevalence of frailty was not different between genders in the univariate analysis (*P* = 0.063), but after adjusting for age, the analysis showed that frailty prevalence was higher among women (OR = 1.17, 95%CI = 1.01–1.36, adjusted *P* = 0.039). Those who had a higher educational level had half the risk of frailty compared with those who were illiterate. The associations between marital status and work status and frailty disappeared after adjustment for age and gender.

Among lifestyle factors, after adjusting for both age and gender, smoking was not associated with frailty, and those who were assessed as having alcohol dependence had more frailty than nondrinkers (OR = 1.84, 95%CI = 1.33–2.55, adjusted *P* < 0.001). An increased level of MIPA was associated with a reduced risk of frailty (high compared to none: OR = 0.52, 95%CI = 0.43–0.64, adjusted *P* < 0.001; low compared to none: OR = 0.74, 95%CI = 0.63–0.88, adjusted *P* = 0.001). No association was observed between organization participation and frailty.

Both SRH and PWB were significantly associated with frailty after adjustment for age and gender. Those who rated their health as poor had a significant increased risk of frailty (OR = 3.16, 95%CI = 2.70–3.68, adjusted *P* < 0.001) compared with those who rated their health as good. Those who were considered to have a psychological disorder also had a significant increased risk of frailty (OR = 3.69, 95%CI = 2.88–4.72, adjusted *P* < 0.001) compared with those who were not.

### Environmental correlates of frailty

Before any independent variables were added to the model, it indicated that there was a statistical significantly variation in frailty across neighbourhoods (χ2 = 134.91, *P* < 0.001). The interclass correlation coefficient (ICC) was 0.127, indicating that 12.7% of the variance in the prevalence of frailty was attributable to neighbourhood-level grouping.

The results of the multilevel model of frailty are presented in Table 4. With only individual-level environmental variables adding to the model (**Model 1**), it indicated negative associations between individual-level AQ and individual-level SP and frailty. For instance, compared to respondents who perceived their neighbourhood’s AQ as being in the first quartile, the OR of frailty for those in the fourth quartile was 0.65 (95%CI: 0.47–0.89). However, with only neighbourhood-level environmental variables adding to the model (**Model 2),** it indicated that there were no significant associations between the four neighbourhood-level environmental characteristics and frailty.

**Table 4:** The Odds Ratios for Frailty associated with Environmental Characteristics (*N* = 2154)

Variable	Model 1			Model 2			Model 3^a^		
	OR	95%CI	*P*	OR	95%CI	*P*	OR	95%CI	*P*
Fixed effects									
Individual-level environment characteristics									
Aesthetic Quality									
1st quartile	1.00						1.00		
2nd quartile	0.79	0.61–1.03	0.077				0.78	0.60–1.02	0.070
3rd quartile	0.79	0.60–1.04	0.093				0.78	0.59–1.02	0.074
4th quartile	0.65	0.47–0.89	0.008				0.65	0.47–0.89	0.008
Walking Environment									
1st quartile	1.00						1.00		
2nd quartile	1.12	0.86–1.47	0.394				1.15	0.88–1.51	0.310
3rd quartile	1.21	0.92–1.59	0.170				1.29	0.97–1.70	0.075
4th quartile	1.13	0.82–1.55	0.451				1.22	0.89–1.67	0.225
Social Cohesion									
1st quartile	1.00						1.00		
2nd quartile	1.02	0.79–1.33	0.863				0.98	0.75–1.28	0.888
3rd quartile	0.91	0.69–1.19	0.485				0.86	0.66–1.14	0.291
4th quartile	0.76	0.56–1.02	0.069				0.73	0.54–0.99	0.043
Social Participation									
1st quartile	1.00						1.00		
2nd quartile	0.76	0.59–0.96	0.025				0.76	0.59–0.97	0.028
3rd quartile	0.60	0.47–0.78	< 0.001				0.59	0.45–0.77	< 0.001
4th quartile	0.61	0.47–0.80	< 0.001				0.59	0.45–0.77	< 0.001
Neighborhood-level environment characteristics									
Aesthetic Quality									
1st quartile				1.00			1.00		
2nd quartile				1.36	0.68–2.73	0.388	1.37	0.69–2.71	0.367
3rd quartile				1.05	0.52–2.13	0.884	1.16	0.58–2.32	0.675
4th quartile				1.32	0.58–2.97	0.506	1.61	0.72–3.61	0.243
Walking Environment									
1st quartile				1.00			1.00		
2nd quartile				1.02	0.48–2.12	0.974	0.97	0.47–2.01	0.945
3rd quartile				0.86	0.42–1.79	0.693	0.81	0.39–1.66	0.561
4th quartile				0.48	0.21–1.07	0.073	0.43	0.19–0.95	0.036
Social Cohesion									
1st quartile				1.00			1.00		
2nd quartile				1.14	0.62–2.09	0.677	1.18	0.65–2.13	0.593
3rd quartile				1.14	0.64–2.02	0.653	1.17	0.67–2.04	0.582
4th quartile				0.63	0.32–1.24	0.181	0.73	0.37–1.42	0.355
Social Participation									
1st quartile				1.00			1.00		
2nd quartile				0.67	0.39–1.14	0.140	0.80	0.46–1.38	0.418
3rd quartile				0.78	0.47–1.32	0.359	1.00	0.60–1.68	0.994
4th quartile				0.92	0.52–1.65	0.784	1.30	0.72–2.34	0.384
Random effects									
Neighborhood-level variance (SE)	0.38 (0.11)			0.24 (0.07)			0.22 (0.07)		
Model fit									
-2LL	4230.3178			4246.6448			4149.2458		
AIC	4260.3180			4276.6450			4227.2460		

^a^gender, age, education, marital status, work status, smoking, and drinking were adjusted

With controlling for individual covariates, and both individual-level and neighbourhood-level variables were added to the final model (**Model 3**), which showed the similar results. Individual-level AQ, individual-level SC, individual-level SP, and neighbourhood-level WE were negatively associated with frailty. Specifically speaking, compared to respondents who perceived the AQ and SC of their neighbourhood to be in the first quartile, the ORs of frailty for those in the fourth quartile were 0.65 (95%CI: 0.47–0.89) and 0.73 (95%CI: 0.54–0.99), respectively. Compared to respondents in the first quartile of SP, the ORs of frailty for those in the second, third and fourth quartiles of SP were 0.76 (95%CI: 0.59–0.97), 0.59 (95%CI: 0.45–0.77) and 0.59 (95%CI: 0.45–0.77), respectively. Compared to the WE of neighbourhoods in the first quartile, the OR of frailty for those in the fourth quartile was 0.43 (95%CI: 0.19–0.95).

## Discussion

The findings of this study showed that the prevalence of frailty among community-dwelling older people in the current sample was similar to previous studies that used the same tool [29, 33]. Plenty of studies have shown that frailty increases with age [2], which was also shown in the current study. Frailty reflects a dynamic health condition; to understand the prevalence and correlates of frailty are important for prevention and intervention policies. This study also indicated that the prevalence of frailty was higher among women and those with a lower educational level, lower moderate intensity physical activity, and poor self-rated health, which is consistent with previous studies [3, 9, 34]. Furthermore, this study provided some different results regarding the risk factors of frailty. Alcohol consumption was associated with many health conditions, but the impact on frailty was absent. Those who are alcohol dependent might not just have a high alcohol consumption; more importantly is the influence on their spirit. Depressive syndrome has had a significant influence on frailty in the previous literature [35, 36], and we found that the influence of psychological well-being on the level of frailty should not be overlooked.

The present study showed the relationship between the physical and social environmental characteristics of the neighbourhood and frailty among community-dwelling older people in Shanghai, China. The final model showed that the individual-level AQ and neighbourhood-level WE of the neighbourhood were negatively associated with frailty by adjusting for individual covariates. Malnutrition (both undernutrition and obesity) plays a key role in the pathogenesis of frailty [37]. The aesthetic quality of the neighbourhood environment has been shown to affect health behaviours such as improving fruit and vegetable consumption, which is an essential component of a healthy diet and one of the most modifiable risk factors for chronic disease [12]. In addition, neighbourhood aesthetics and walking environment were correlated with obesity and body mass index (BMI) [13, 38]. Even more, a high aesthetic quality of the neighbourhood probably improves residents’ pleasant mood, and was related to a higher mental well-being [22, 39] and lower depressive symptoms [40]. The walkability of the neighbourhood refers to walkable roads, access to facilities and opportunities for activities, which are correlated with various types of physical activity [13, 23] and chronic diseases [41, 42]. Older people living in a more walkable neighbourhood seem to have more opportunities to get out and interact with others, even residents with mobility impairment [13].

Our study showed that individual-level SC and individual-level SP were negatively associated with frailty. A previous study found that in community-dwelling older people, a stronger sense of social cohesion (refers to trust and interaction with neighbours) seemed to protect against frailty [25]. In our study, SC was measured by helping neighbours, getting along with each other, trust, and sharing the same values. People who perceived their neighbourhoods as more cohesive might be more likely to improve the positive social norms regarding healthy behaviours and social support. Neighbours who trust each other are more likely to provide help with promoting access to services and amenities in daily life. A systematic review highlighted that social participation had more consistent results than other social environmental characteristics for predicting frailty [24]. Not like SC, which refers to inter-related features of society, SP focus more on individual participation in several social activities within daily life. In China, most older adults retire at retirement age or do not have a formal job beforehand, and becoming involved in neighbourhood activities is very common for them. Previous studies found a relationship between social participation (frequent group engagement, attending a religious service, physical leisure activities, participation in helping other people) and lower frailty [43, 44]; and another study also showed evidence that both individual-level and community-level sports group participation and individual-level hobby group participation were positively correlated with physical and mental health among older adults [11, 45]. In the current study, SP included eight types of participation. To be specific, these neighbourhood social activities involved helping manage chronic diseases, accessing health knowledge, increasing physical activities and promoting interpersonal relationships, which are all in favour of health. Those who were more likely to participate in those activities regularly gained more health benefits.

There are some limitations to our study. First, the cross-sectional study design could not define the direction of causality. Second, neighbourhood-level environmental characteristics were measured by self-reported questionnaires rather than objective measures, and therefore cannot represent the true conditions of the neighbourhood environment. In the future, a longitudinal study with an objective neighbourhood environmental characteristics measurement, more balanced and representative samples and that controls for more confounding variables (for example socio-economic characteristics) should be conducted to explain the relationships between individual factors and neighbourhood environmental characteristics and frailty among community-dwelling older people.

## Conclusions

This study indicates that frailty is a highly prevalent health condition among the aged population in China. Both individual factors and environmental characteristics of the neighbourhood are associated with frailty among Chinese community-dwelling older people. Primary healthcare should pay more attention to those who are older age, women, those with a low educational level, and those with mental health problems among the frail elderly. It is important to encourage healthy behaviours and social participation; meanwhile, building aesthetic, walkable and cohesive neighbourhoods may decrease frailty among Chinese community-dwelling older people.

## Additional file

Additional file 1: The dataset of analysis. The dataset of analysis comes from a subsample of the Shanghai Healthy City Survey (Round 5). It includes the total complete data (2559 elders) in Sheet 1 and the processed data for multilevel analysis (2154 elders) in Sheet 2. (XLSX 1262 kb)

## Abbreviations

-2LL: -2 log likelihood
AIC: Akaike information criterion
ANOVA: Analysis of variance
AQ: Aesthetic quality
BMI: Body mass index
CAGE: Cut down, Annoyed, Guilty, Eye opener
CI: Confidence interval
FI: Frailty index
FP: Frailty phenotypes
FRAIL: Fatigue, Resistance, Ambulation, Illness, Loss of weight
MIPA: Moderate intensity physical activity
OR: Odds ratio
SC: Social cohesion
SD: Standard deviation
SE: Standard error
SP: Social participation
SPSS: Statistical Package for the Social Sciences
WE: Walking environment
WHO: World Health Organization
